# Supplementary figures and images for: Phylogeography of Rotavirus G8P[8] Detected in Argentina: Evidence of Transpacific Dissemination
Source: Viruses. 2022 Oct 9;14(10):2223. doi: 10.3390/v14102223 (PMC9609476; doi:10.3390/v14102223)

country

- ARG
- CHL
- CHN
- CZE
- EGY
- IND
- JPN
- SGP
- SKO
- THA
- VNM

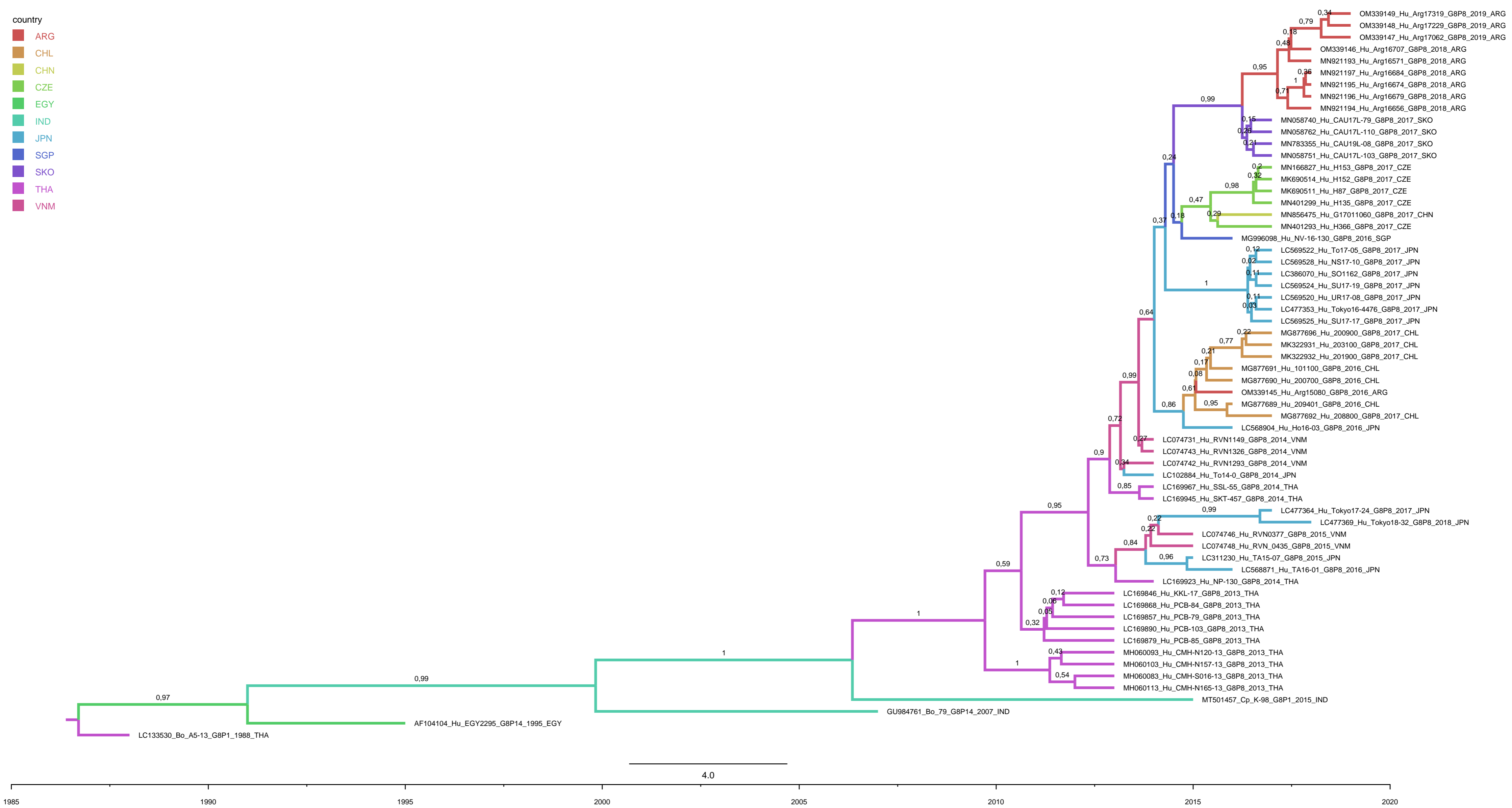

Supplement: Supplementary file 1 [file viruses-14-02223-s001.zip › G8_LineageIV_MCCT_CladePosteriorProbability.pdf]

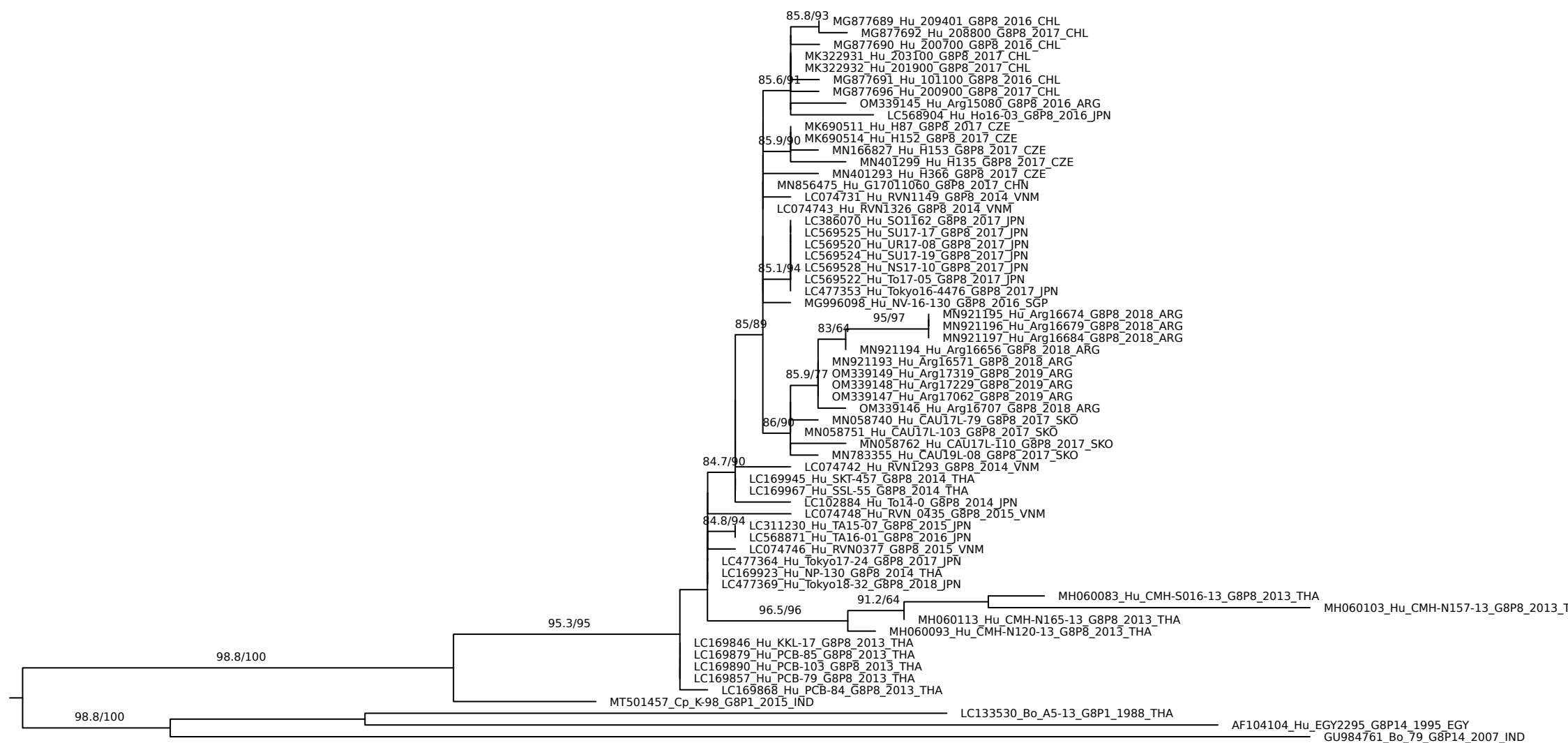

0.01

Supplement: Supplementary file 1 [file viruses-14-02223-s001.zip › G8P8_LineageIV_MLtree.pdf]
